# Degenerative Mitral Regurgitation Outcomes in Asian Compared With European-American Institutions

**DOI:** 10.1016/j.jacasi.2024.03.003

**Published:** 2024-05-21

**Authors:** Nadira Hamid, Francesca Bursi, Giovanni Benfari, Jean-Louis Vanoverschelde, Christophe Tribouilloy, Elena Biagini, Jean-Francois Avierinos, Andrea Barbieri, Yiting Fan, Federico Guerra, Chua Yeow Leng, Benjamin Essayagh, Agnés Pasquet, Catherine Szymansky, Alexis Théron, Hector I. Michelena, Vuyisile T. Nkomo, David Vancraeynest, Dan Rusinaru, Francesco Grigioni, Maurice L. Enriquez-Sarano, Ding Zee Pin, Alex Pui-Wai Lee, the MIDA Investigators

**Affiliations:** a National Heart Centre Singapore, Singapore; b New York Presbyterian Hospital, Columbia University Medical Center, New York, New York; c University of Milan, Department of Health Sciences, Division of Cardiology, San Paolo Hospital, ASST Santi Paolo e Carlo, Milan, Italy; d Department of Cardiovascular Medicine, Mayo Clinic, Rochester Minnesota, USA; e University of Verona, Department of Medicine, Section of cardiology, Verona, Italy; f Pôle de Recherche Cardiovasculaire, Institut de Recherche Expérimentale et Clinique, Université Catholique de Louvain and Divisions of Cardiology and Cardiothoracic Surgery, Cliniques Universitaires Saint-Luc, Brussels, Belgium; g Department of Cardiology, Amiens University Hospital, Amiens, France, and EA 7517 MP3CV Université de Picardie Jules Verne University Hospital, Amiens, France; h Cardiovascular Department, University Hospital S. Orsola-Malpighi, Bologna, Italy; i Cardiovascular Division, Aix-Marseille Université, INSERM MMG U1251, Marseille, France; j Divison of Cardiology, Department of Diagnostics, Clinical and Health Public, University of Modena, University of Modena and Reggio Emilia, Modena, Italy; k Shanghai Chest Hospital, Shanghai, P.R. China; l Cardiology and Arrhythmology Clinic, Marche Polytechnic University, University Hospital "Umberto I - Lancisi - Salesi", Ancona, Italy; m Cardiovascular Department, University Campus Bio-Medico, Rome, Italy; n Minneapolis Heart Institute, Minneapolis, Minnesota, USA; o Li Ka Shing Institute of Health Sciences, The Chinese University of Hong Kong, Sha Tin, Hong Kong, P.R. China

**Keywords:** degenerative mitral regurgitation, ethnicity, mitral surgery, outcome

## Abstract

**Background:**

Clinical outcome and interventional thresholds for degenerative mitral regurgitation (DMR) were developed in studies of patients at European and American institutions (EAIs), but little is known about patients at Asian institutions (AsIs).

**Objectives:**

This study sought to contrast DMR presentation/management/outcomes of AsI patients vs EAI patients.

**Methods:**

Patients with DMR due to flail leaflet from Hong Kong and Singapore (AsI cohort, n = 737) were compared with EAI patients (n = 682) enrolled in the MIDA (Mitral regurgitation International Database) registry with similar eligibility criteria.

**Results:**

AsI patients presented similar DMR lesion/consequences vs EAI patients, but they were younger, with fewer symptoms (74% vs 44% Class I), more sinus rhythm (83% vs 69%), and lower EuroSCORE II (European System for Cardiac Operative Risk Evaluation II) (0.9 ± 0.5 vs 1.4 ± 1.5; all *P* < 0.0001). Imaging showed smaller absolute left atrial/ventricular dimensions in AsI patients, belying cardiac dilatation with larger body surface area–indexed diameters (all *P* < 0.01). Surgical/interventional mitral repair was similarly predominant (90% vs 91%; *P* = 0.47), and early repair was similarly beneficial (for AsI patients, adjusted HR: 0.28; 95% CI: 0.16-0.49; for EAI patients, HR: 0.32; 95% CI: 0.20-0.49; both *P* < 0.0001). However, AsI patients underwent fewer interventions (55% ± 2% vs 77% ± 2% at 1 year; *P* < 0.0001) and incurred excess mortality (adjusted HR: 1.60 [95% CI: 1.13-2.27] vs EAI patients; *P* = 0.008) at long-term postdiagnosis. Propensity score matching (434 patient pairs), which balanced all clinical characteristics, confirmed that there was undertreatment and excess mortality in the long term in AsI patients with DMR (*P* < 0.0001).

**Conclusions:**

Imaging may underestimate volume overload in AsI patients due to smaller cardiac cavities related to smaller body size compared with EAI patients with similar mitral lesions and DMR severity. AsI patients enjoy similar mitral repair predominance and early intervention benefits but undergo fewer mitral interventions than EAI patients and incur subsequent excess mortality, suggesting the need to account for imaging and cultural specificity to improve DMR outcomes worldwide.

Degenerative mitral regurgitation (DMR) is a progressive disease and when severe is associated with excess mortality and morbidity with medical management.^1^, ^2^, ^3^, ^4^ Recent studies in Europe and the United States have demonstrated a disturbing trend toward undertreatment, even in symptomatic patients and in major institutions.^3^^,^^5^ These clinical registries of DMR outcomes in routine clinical practice have provided important support for guideline criteria/thresholds^6^^,^^7^ and emphasized the profound impact of mitral repair^8^ and early repair^9^ approaches on clinical outcomes.

However, there are important and unresolved issues regarding DMR among patients diagnosed in Asia. Indeed, European and U.S. guidelines include left ventricular end-systolic diameter (LVESD) as a Class I criterion for surgery, but only as absolute dimension without accounting for body size, which hinders the care of women with DMR,^10^ and may be relevant to our patients of generally smaller body sizes in Asia. Furthermore, for DMR management and outcome, most studies were conducted in Europe and the United States,^9^^,^^11^ with little information on clinical management and outcomes of DMR in cohorts recruited from Asia. While benefits of early DMR repair in Asian observational studies for patients older than 50 years of age^12^ appear similar to U.S. and European Union patients, whether these aggressive therapeutic approaches are implemented in routine practice is uncertain. More generally, studies describing DMR imaging features, management, and outcomes in Asian populations are lacking.^9^^,^^13^, ^14^, ^15^, ^16^ Hence, the pertinence of current guidelines to patients diagnosed in Asia remains uncertain and warrants careful assessment to ensure that clinical recommendations are applicable to worldwide populations for optimal outcomes.

To address these gaps in knowledge, we gathered from our practices 2 large populations of patients with DMR due to flail leaflet from Hong Kong and Singapore, summarily called the Asian institution (AsI) cohort. To provide a comparison for our patients, we obtained control subjects with similar DMR and similar definition of valve lesion enrolled in the largest international multicenter European and American institutions (EAIs) (ie, the MIDA [Mitral regurgitation International Database] registry). Thus, these cohorts provide similarly defined DMR patients, enrolled by similarly trained physicians, within EAI countries of similar economic status/standards of living/access to treatment akin to those in Hong Kong and Singapore. We aimed at comparing imaging features, management, and outcomes of patients in our AsIs vs those in EAIs, diagnosed with similar DMR.

## Methods

### Study design

Patients with DMR recruited in 1 center in Singapore (National Heart Centre Singapore) and 1 center in Hong Kong (Prince of Wales Hospital, the Chinese University of Hong Kong) were compared with patients with a similar definition of DMR due to flail leaflet enrolled in the MIDA registry. Both hospitals in Hong Kong and Singapore are tertiary institutions providing health care services to over 40% of the local Asian populations affected by DMR in their respective regions.^17^^,^^18^ The MIDA registry systematically merged the consecutive experience with patients diagnosed with DMR due to flail mitral leaflets in tertiary centers: 2 in France (university hospitals in Amiens and Marseille), 3 in Italy (university hospitals in Bologna, Ancona, and Modena), 1 in Belgium (university hospital in Brussels), and 1 in the United States (Mayo Clinic in Rochester, Minnesota). All centers provided approval from ethics boards and Institutional Review Boards, which waived the informed consent requirement in some centers. Specifically, as prescribed by European law, no ethnic/racial identification was abstracted. Patients were identified retrospectively, and their clinical data, stored in the clinical and echocardiographic repositories, were accessed electronically without alterations.

### Patient population

The inclusion/exclusion criteria of the AsI cohort and the MIDA registry were similar and have been previously described (Supplemental Appendix).^2^^,^^9^^,^^11^^,^^19^ For the present study, to improve enrollment homogeneity between the AsI and EAI cohorts, the enrollment period was set between January 1, 2005, and December 31, 2014, for both cohorts.

### Echocardiography

Transthoracic echocardiography was performed in routine clinical practice in each academic center, and measurements were guided by American Society of Echocardiography guidelines as previously described (Supplemental Appendix).^20^^,^^21^

### Follow-up and outcome measures

The endpoints collected were: 1) performance/type of interventional/surgical treatment of DMR; and 2) occurrence of death, overall after diagnosis and postoperatively. Patients were followed by their primary physicians at participating or referral institutions. Data were collected through direct review of clinical records, patient interviews, and/or follow-up letters and questionnaires. Follow-up data collection up to 2019 was complete for >95% of enrolled patients in each center.

### Statistical analysis

Data are presented as mean ± SD, median (Q1-Q3), or percentage as appropriate. Group comparisons used Student’s *t* test, the Mann Whitney *U* test, or the chi-square test, as appropriate.

Outcomes were displayed using the Kaplan-Meier method for incidence of mitral intervention and for survival including postoperative survival and compared using the log-rank test. Age-adjusted survival curves obtained by direct adjustment to the age at diagnosis were compared between AsI patients and EAI patients. To analyze the impact of mitral interventions on survival, landmark analysis and compared early mitral intervention (within 3 months of diagnosis) vs medical management, excluding patients deceased or censored prior to the 3-month landmark.^22^^,^^23^ Mitral interventions’ impact on outcome was also analyzed as a time-dependent variable within the entire follow-up. In view of expected baseline differences between cohorts, multivariable Cox proportional hazards models with comprehensive adjustment determined the independent associations of patients’ institutional origin (AsIs vs EAIs) with endpoints of mitral intervention and survival including postoperative survival. To ensure balance of clinical baseline characteristics between cohorts, we also performed greedy nearest-neighbor propensity score matching using logistic regression between AsI patients and EAI patients (Supplemental Appendix) and then compared the matched subcohorts for clinical outcome. Clinical outcome differences are presented as HRs between AsI patients and EAI patients with 95% CIs. Data were analyzed with the SPSS 23 (IBM), SAS and JMP 14 (SAS Institute), and MedCalc version 16.2.0 (MedCalc) software packages.

## Results

### Baseline characteristics

In the selected time frame, 737 patients were identified from AsIs and 682 patients were obtained for comparison from EAIs, providing a total of 1,419 patients with DMR due to flail leaflet.

Overall baseline characteristics (Table 1) were typical of DMR (age 63 ± 13 years, male 72%), were mostly asymptomatic or mildly symptomatic at diagnosis, and were with the flail leaflet mostly affecting the posterior leaflet causing mostly severe DMR with dilated left ventricle (LV) and left atrium (LA).

**Table 1:** Baseline and Echocardiographic Characteristics of the Study Population

	Total (N = 1,419, 100%)	AsI Patients (n = 737, 52%)	EAI Patients (n = 682, 48%)	*P* Value
Clinical characteristics				
Age at diagnosis, y	63 ± 13	61 ± 12	66 ± 14	<0.0001
Men	72	71	74	0.20
NYHA functional class				
I	844 (60)	544 (74)	300 (44)	<0.0001
II	401 (28)	120 (16)	281 (41)	
III	140 (10)	61 (8)	79 (12)	
IV	32 (2)	10 (1)	22 (3)	
Sinus rhythm at diagnosis	1,079 (76)	609 (83)	470 (69)	<0.0001
Hypertension	642 (46)	388 (54)	254 (37)	<0.0001
Diabetes mellitus	142 (10)	85 (12)	57 (8)	0.02
Dyslipidemia	488 (35)	251 (35)	237 (35)	0.80
History of CAD	377 (27)	162 (22)	215 (32)	<0.0001
EuroSCORE II	1.13 ± 1.2	0.9 ± 0.5	1.4 ± 1.5	<0.0001
Morphometric characteristics				
Weight, kg	72 ± 19	64 ± 17	80 ± 18	<0.0001
Height, cm	168 ± 11	163 ± 9	174 ± 10	<0.0001
Body surface area, m^2^	1.8 ± 0.3	1.7 ± 0.2	1.9 ± 0.2	<0.0001
Heart rate, beats/min	76 ± 18	88 ± 23	73 ± 15	<0.0001
LA diameter, mm	48 ± 9	48 ± 12	48.9 ± 5	0.03
Indexed LA diameter, mm/m^2^	27 ± 7	29 ± 8	26 ± 4	<0.0001
LVEDD, mm	55 ± 8	52 ± 8	58 ± 7	<0.0001
Indexed LVEDD, mm/m^2^	31 ± 5	31 ± 6	30 ± 4	0.001
LVESD, mm	35 ± 8	33 ± 9	36 ± 7	<0.0001
Indexed LVESD, mm/m^2^	20 ± 5	20 ± 6	19 ± 3	<0.0001
LVEF, %	62 ± 9	62 ± 9	63 ± 9	<0.0001
Ventricular septal thickness, mm	10 ± 2	10 ± 2	11 ± 2	<0.0001
LV mass, g	229 ± 88	207 ± 94	255 ± 71	<0.0001
Indexed LV mass, g/m^2^	127 ± 45	123 ± 54	131 ± 31	0.002
RVSP, mm Hg	42 ± 17	42 ± 17	42 ± 17	0.70
Severe mitral regurgitation	1,226 (88)	619 (86)	607 (89)	0.10
Isolated anterior leaflet flail	196 (14)	107 (15)	88 (13)	0.20
Isolated posterior leaflet flail	1,132 (80)	610 (83)	522 (77)	0.004

Values are mean ± SD, %, or n (%).

AsI = Asian institution; CAD = coronary artery disease; EAI = European and American institution; EuroSCORE II = European System for Cardiac Operative Risk Evaluation II; LA = left atrial; LV = left ventricular; LVEDD = left ventricular end-diastolic dimension; LVEF = left ventricular ejection fraction; LVESD = left ventricular end-systolic dimension; RVSP = right ventricular systolic pressure.

Comparison of AsI patients and EAI patients (Table 1) showed profound (anterior flail location, DMR severity, systolic pulmonary artery pressure; all *P* > 0.10) or close (posterior flail location) imaging similarities of valvular lesions/consequences. Similarly, DMR quantitation (performed when judged necessary: 191 AsI patients, 399 EAI patients) showed close regurgitant volume (83 ± 28 mL/beat vs 90 ± 49 mL/beat; *P* > 0.10), well within the severe DMR range. Clinically, male predominance was identical in both populations, but AsI patients were younger with different distributions of risk factors and associated conditions. Consistent with younger age at diagnosis, the burden of comorbidity was lower (lower EuroSCORE II [European System for Cardiac Operative Risk Evaluation II]) and more patients were asymptomatic or in sinus rhythm among AsI patients (all *P* < 0.0001). Morphometric data showed considerably different body habitus with smaller height and weight in AsI patients, resulting in smaller body surface area than EAI patients (1.7 ± 0.2 m^2^ vs 1.9 ± 0.2 m^2^; *P* < 0.0001). Imaging showed also considerable morphometric differences at diagnosis: LV and LA sizes were much smaller in AsI patients, while the difference in left ventricular ejection fraction (LVEF) was statistically but not clinically significant. Thus, LVESD ≥40 mm was observed in 16% of AsI patients vs 24% of EAI patients (*P* < 0.001), and LV mass was also smaller in AsI patients. Nevertheless, after normalizing to body surface area, indexed LV and LA sizes were larger in AsI patients; the LV mass index difference was greatly attenuated (Central Illustration). Hence, the extent of cardiac remodeling related to DMR was significantly underestimated in AsI patients. However, taking into account smaller body size of AsI patients, it became evident that these patients experienced a substantial volume overload.

**Central Illustration:**
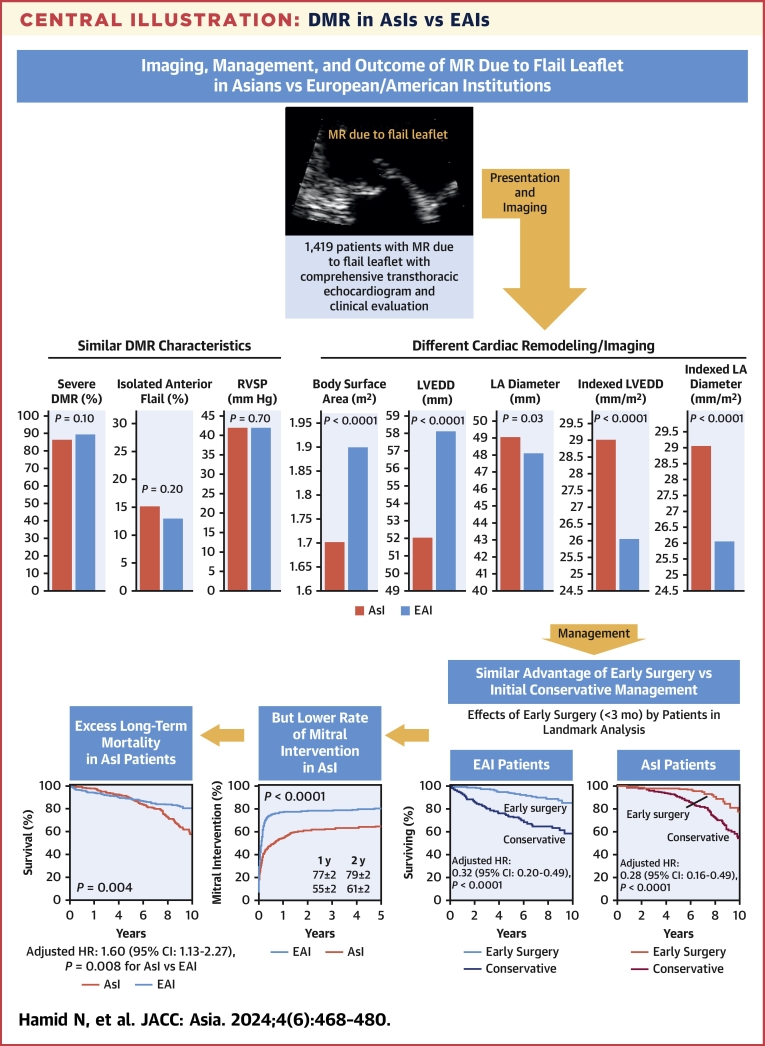
DMR in AsIs vs EAIs Imaging diagnosis of degenerative mitral regurgitation (DMR) due to flail mitral leaflet (top left) is similar with similar flail location and DMR severity, but striking differences at presentation mostly relate to smaller body size in Asian institutions (AsIs) (red) vs European and American institutions (EAIs) (blue) centers (top right, 2 bar graphs) that may lead to underestimation of volume overload. The impact of early mitral intervention on survival (lower row, right) is similar, but rates of mitral interventions in Asians (lower row, middle) are markedly lower. The long-term outcome shows age-adjusted excess mortality in Asians (lower row, left). LA = left atrial; LVEDD = left ventricular end-diastolic dimension; LVEF = left ventricular ejection fraction; MR = mitral regurgitation; RVSP = right ventricular systolic pressure.

### Clinical outcomes

#### Mitral valve interventions after diagnosis

During follow-up, 1,012 patients underwent mitral valve intervention, 65% among AsI patients and 79% among EAI patients (*P* < 0.0001). The median time from diagnosis to intervention was 42 days (Q1-Q3: 7-183 days) in AsI patients vs 24 days (Q1-Q3: 3-61 days) in EAI patients (*P* < 0.0001). The proportion of patients undergoing mitral surgery/intervention who received mitral repair was high and was similar in AsI and EAI patients (90% vs 91%; *P* = 0.47), including 10 AsI patients who underwent percutaneous edge-to-edge-repair.

**Figure 1:**
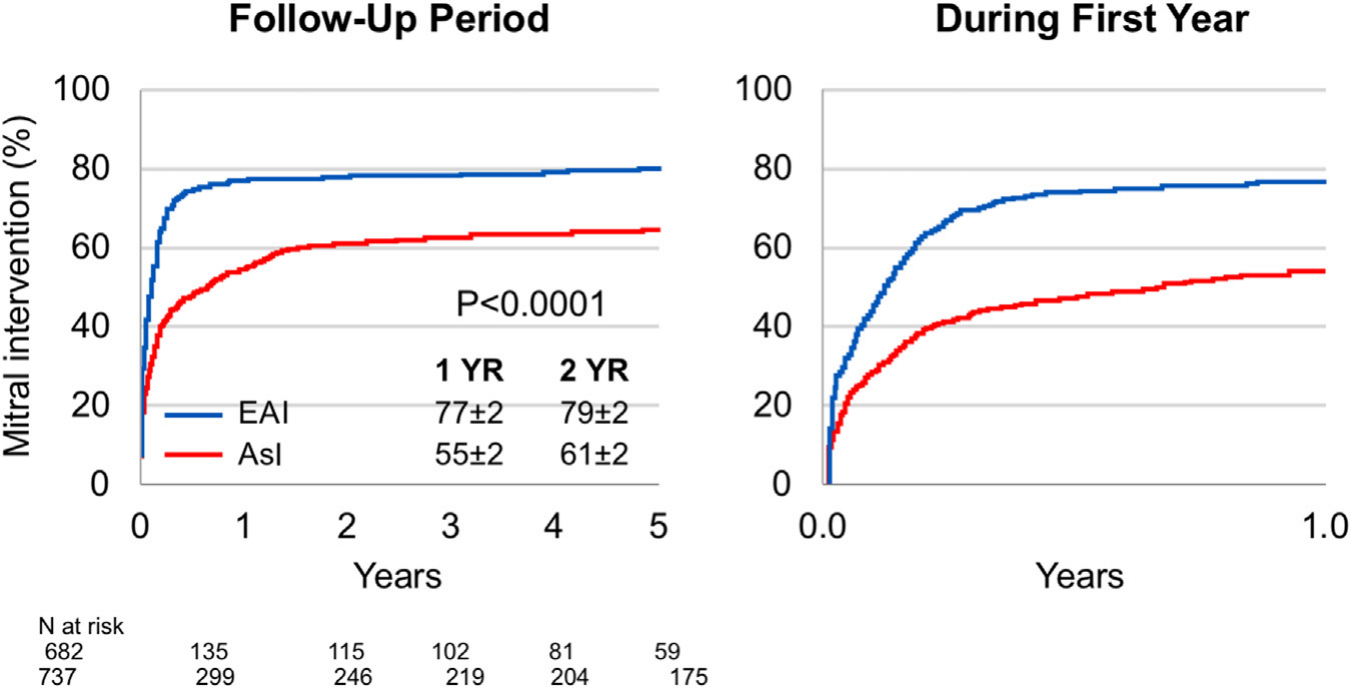
Cumulative Incidence of Mitral Valve Intervention Rates of mitral intervention after diagnosis indicated over the first 5 years of follow-up (left) and expanded during the first year postdiagnosis (right). Note that among Asians there were fewer mitral interventions, particularly in the first year postdiagnosis, with the later interventional rate flatter than in Westerners. AsI = Asian institutions; EAI = European and American institutions.

Figure 1 shows cumulative incidence of mitral intervention, which was lower in AsI patients than EAI patients at all times and was delayed (*P* < 0.0001). Throughout the entire follow-up, AsI patients had a 41% lower probability of undergoing mitral valve intervention (HR: 0.59; 95% CI: 0.52-0.67; *P* < 0.0001). This association persisted after multivariable adjustment (Table 2), including comprehensive adjustment (adjusted HR: 0.50; 95% CI: 0.42-0.47; *P* = 0.0001).

**Table 2:** Relative Risk of Mitral Valve Intervention and Overall Death for AsI Patients vs EAI Patients

	Mitral Intervention		Overall Death	
	HR (95% CI)	*P* Value	HR (95% CI)	*P* Value
Model 1 (unadjusted)	0.58 (0.51-0.66)	0.0001	0.85 (0.65-1.10)	0.21
Model 2 (adjusted for age, sex, EuroSCORE II)	0.50 (0.44-0.57)	0.0001	1.40 (1.05-1.85)	0.02
Model 3 (adjusted for age, sex, EuroSCORE II, NYHA, sinus rhythm, history of CAD)	0.55 (0.48-0.62)	0.0001	1.56 (1.17-2.10)	0.003
Model 4 (adjusted for age, sex, EuroSCORE II, NYHA, sinus rhythm, history of CAD, LVEF, indexed LVESD, RVSP)	0.50 (0.42-0.47)	0.0001	1.60 (1.13-2.27)	0.008

Abbreviations as in Table 1.

**Figure 2:**
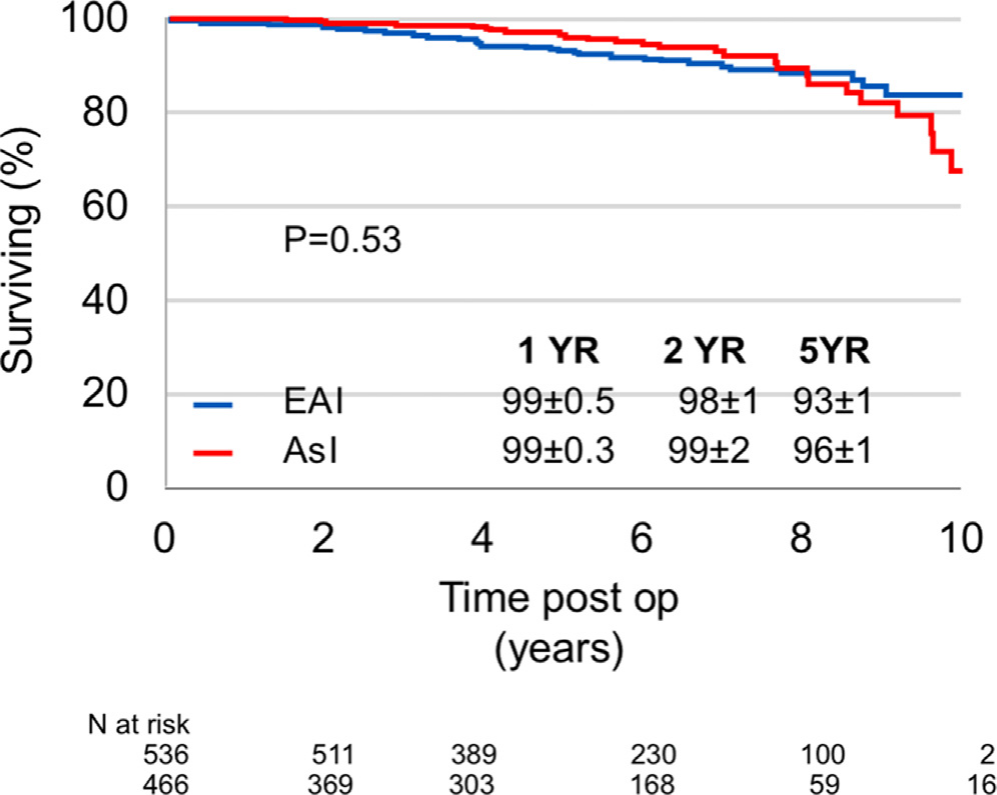
Postoperative Survival After Mitral Valve Intervention Survival rates after mitral interventions are similar in Asians and Westerners and remain so even after comprehensive adjustment. Abbreviations as in Figure 1.

In patients who underwent mitral interventions, 76 postoperative deaths were recorded, with similar rates of post–mitral intervention mortality in AsI and EAI patients (Figure 2) even after adjustment for age, sex, and EuroSCORE II (adjusted HR: 1.46; 95% CI: 0.88-2.42; *P* = 0.14).

#### Association of early mitral intervention and mortality

**Figure 3:**
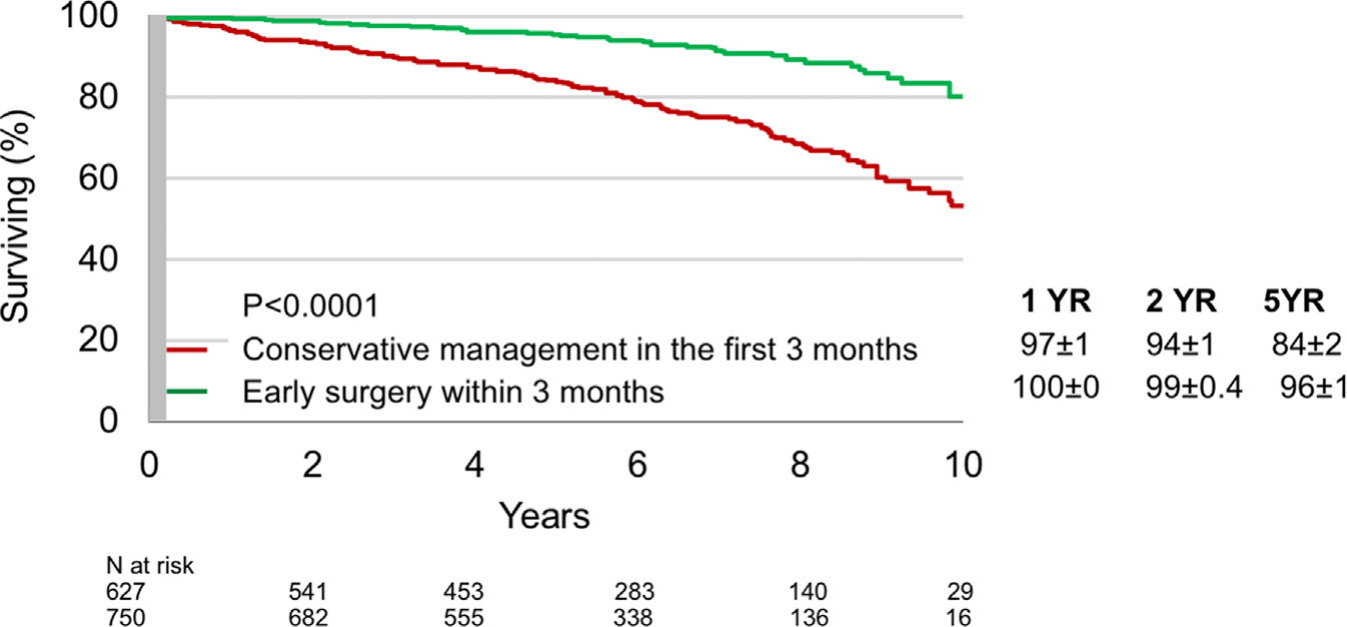
Effect of Early Surgery and Long-Term Survival in Landmark Analysis Survival beyond the first 3 months postdiagnosis according the treatment indicated during the first 3 months. The gray shaded area marks the landmark time set at 3 months from diagnosis. Note the markedly superior survival after early surgery that persists even after comprehensive adjustment.

**Figure 4:**
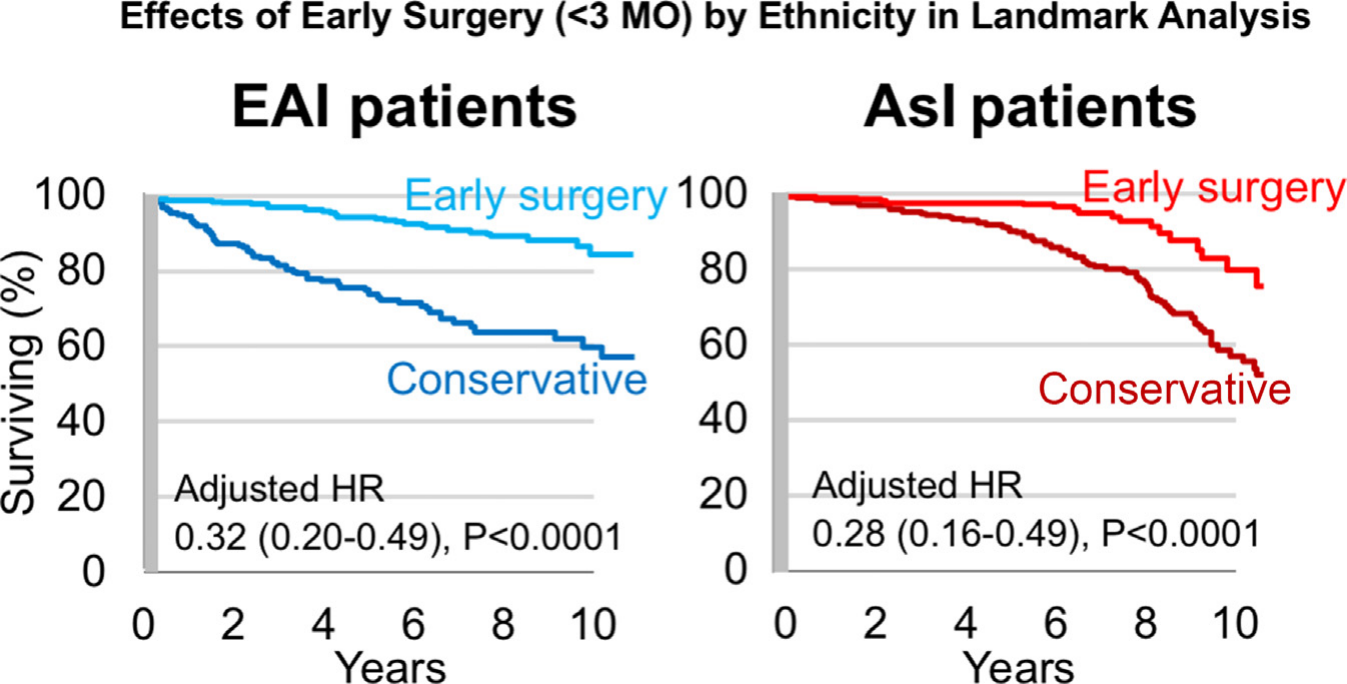
Survival by Early Surgery Stratified by Origin in Landmark Analysis Survival beyond the first 3 months postdiagnosis is presented comparing those receiving early surgery with those followed conservatively during the first 3 months postdiagnosis in Western (left) and Asian (right) institutions. The gray shaded area marks the landmark time set at 3 months from diagnosis. Note that the survival benefit associated with early surgery is similar in both cohorts. The dark red line indicates conservative. Abbreviations as in Figure 1.

Landmark analysis was performed to evaluate the effect of early intervention (<3 months) compared with initial conservative management on overall survival (Figure 3). Five years after diagnosis, survival was much higher after early intervention than with initial conservative management (96% ± 1% vs 84% ± 2%; *P* < 0.0001). Both in the overall cohort and in AsI patients and EAI patients separately, the beneficial effect of early intervention was maintained throughout follow-up (Figure 4). After adjustment for age, sex, EuroSCORE II, institutional origin (AsI vs EAI), NYHA functional class, sinus rhythm, coronary artery disease history, LVEF, indexed LVESD, and right ventricular systolic pressure, mitral intervention as a time-dependent variable was associated with reduced mortality (adjusted HR: 0.47; 95% CI: 0.32-0.68; *P* < 0.0001) confirming the benefit of mitral surgical/interventional treatment. This strong association was maintained after stratification by origin (Table 3), in AsI patients (adjusted HR: 0.26; 95% CI: 0.14-0.47; *P* < 0.0001) and in EAI patients (adjusted HR: 0.24; 95% CI: 0.15-0.39; *P* < 0.0001). Furthermore, early intervention was an independent determinant of survival in comprehensively adjusted multivariable analysis (adjusted HR: 0.27; 95% CI: 0.37-0.53; *P* < 0.0001) without interaction with patients’ origin (*P* = 0.53). All analyses were repeated excluding patients who underwent percutaneous edge-to-edge-repair, and results were unaffected.

**Table 3:** Mitral Intervention Impact in Time-Dependent Analysis on Overall Survival in the Total Cohort and by Origin

	Mitral Interventions as Time-Dependent Exposure					
	Total Cohort		AsI Patients^a^		EAI Patients^a^	
	HR (95% CI)	*P* Value	HR (95% CI)	*P* Value	HR (95% CI)	*P* Value
Model 1 (adjusted for age and sex)	0.32 (0.24-0.43)	<0.0001	0.37 (0.24-0.57)	<0.0001	0.30 (0.20-0.45)	<0.0001
Model 2 (adjusted for age, sex, EuroSCORE II, AsI patients origin)	0.33 (0.23-0.43)	<0.0001	0.34 (0.23-0.55)	<0.0001	0.32 (0.21-0.48)	<0.0001
Model 3 (adjusted for age, sex, EuroSCORE II, AsI patients origin, NYHA functional class, sinus rhythm, history of CAD)	0.31 (0.27-0.43)	<0.0001	0.29 (0.19-0.47)	<0.0001	0.27 (0.18-0.43)	<0.0001
Model 4 (adjusted for age, sex, EuroSCORE II, AsI patients origin, NYHA functional class, sinus rhythm, history of CAD, LVEF, indexed LVESD, RVSP)	0.47 (0.32-0.68)	<0.0001	0.26 (0.14-0.47)	<0.0001	0.24 (0.15-0.39)	<0.0001

Abbreviations as in Table 1.

a Not adjusted for institutional origin. *P* for interaction institutional origin/time to surgery in adjusted models = NS.

### Long-term survival

**Figure 5:**
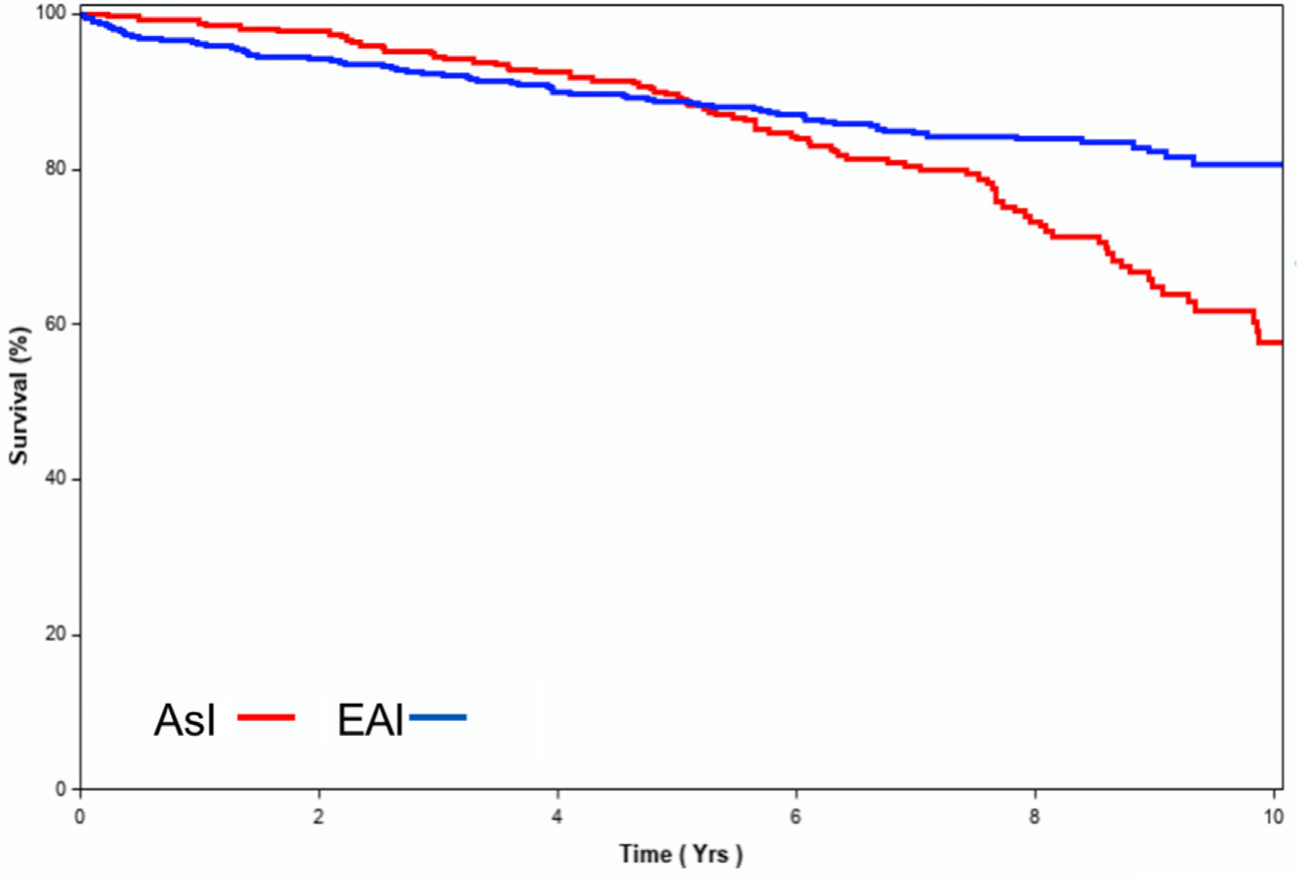
Age-Adjusted Survival After DMR Diagnosis in AsI Patients and EAI Patients The age-adjusted survival after degenerative mitral regurgitation (DMR) diagnosis is similar during the first 5 years of follow-up, but secondarily, high-mortality rates observed in As-I patients yields overall excess mortality in this cohort confirmed in multivariable analysis. Abbreviations as in Figure 1.

During follow-up of 5.4 ± 2.8 years (median 5.5 years [Q1-Q3: 3.6-7.4 years]), 232 deaths were recorded. The overall survival at 5 and 10 years among AsI patients was 93 ± 1% and 60 ± 4%, while among EAI patients, it was 85% ± 1% and 74% ± 3%, respectively (*P* = 0.21). However, age-adjusted survival curves demonstrated excess mortality in AsI patients (likelihood ratio *P* = 0.0042) once the baseline age difference between cohorts was accounted for (Figure 5). Adjustment for age, sex, EuroSCORE II, NYHA functional class, sinus rhythm, prior history of coronary artery disease, LVEF, indexed LVESD, and right ventricular systolic pressure confirmed independent association between AsI patients and overall excess mortality vs EAI patients (Table 2).

To further evaluate the impact of early intervention vs comorbidity on AsI patients excess mortality, we added early intervention to the comprehensive model for overall survival and observed that early intervention not only remained highly significant (*P* < 0.0001), but also reduced the HR attached to AsI patients from 1.60 to 1.15, which became nonsignificant, demonstrating the strong association of low early surgery performance and excess mortality in AsI patients.

### Propensity score-matched cohorts

To examine AsI and EAI with as balanced as possible baseline characteristics, greedy propensity score matching of the AsI and EAI cohorts based on an extensive list of covariates was conducted (Methods and Supplemental Appendix). Matching was highly successful, with 434 patient pairs demonstrating near equalization of baseline variables, particularly age, after matching (Supplemental Figure 1). All clinical characteristics (even those not matched for) displayed no significant differences (Supplemental Table 1), while morphometric data showed the expected persistent body size and absolute heart size differences (Supplemental Table 1). Thus, matched for all clinical characteristics, AsI patients displayed similar morphometric differences (Table 1, Supplemental Table 1), similar undertreatment by mitral interventions (Supplemental Figure 2), and similar excess mortality (Supplemental Figure 3) as the overall cohort. While patients AsI and EAI treated by early surgery tended to be younger and lower risk (Supplemental Table 2) than those remaining under conservative management, comprehensive adjustment showed a similar benefit of early surgery in both subsets (Table 3).

### Patient and public involvement

This was a retrospective evaluation of standard care and routinely collected data. Therefore, no patients or members of the public were directly involved.

## Discussion

By analyzing patients diagnosed with DMR in AsIs and comparing them with a large cohort with the same imaging diagnosis of DMR due to flail leaflets in EAIs with a similar socioeconomic environment, this study provides unique and new insights. Imaging shows striking similarities between AsI patients and EAI patients, in terms of mitral regurgitation cause and mechanism (flail segment) of DMR severity and hemodynamic consequence on pulmonary hypertension. The most striking baseline differences are morphometric, with smaller body size in AsI patients and considerably smaller absolute values of LV and LA dimensions in AsI patients despite similar DMR severity, and far from guideline-based thresholds. However, normalized to body size, the LA and LV were larger in AsI patients, demonstrating volume overload severity and the need to account for body size. In terms of treatment effectiveness, repair interventions predominated similarly, and when performed, provided similar survival benefit in AsI patients and EAI patients. Notwithstanding, we demonstrate for the first time that management is very different in our cohorts of AsI patients, with considerably fewer and delayed mitral interventions compared with EAI patients. Consequently, outcome is ultimately affected, with long-term excess mortality in AsI patients. Multivariable analysis and propensity score matching, adjusting for and balancing clinical characteristics, confirmed both undertreatment and excess mortality in AsI patients. Therefore, DMR imaging and management should account for these specificities and strive toward early mitral interventions and optimal outcomes in patients with DMR worldwide.

### DMR heterogeneity

Little is known on mechanisms of myxomatous degeneration causing DMR and possible population differences. For example, women with mitral valve prolapse present with mitral lesions distributed differently than men.^10^ Despite similar flail mechanism and DMR severity, presentation at younger ages in AsI patients questions potentially faster progressing myxomatous degeneration. The younger age of patients with mitral regurgitation in Asia in various geographic locations^12^ is quite notable vs similar studies in EAI patients.^9^ Previous DMR studies demonstrated distinct genetic expression patterns in mitral tissue,^24^^,^^25^ but contrasting analysis by geographic locations is yet unavailable. Hence, it is currently uncertain whether younger age of AsI patients results from true biological difference, and future studies involving systematic analysis of myxomatous degeneration and mitral prolapse development in population-based settings are warranted. The age difference between AsI patients and EAI patients may reveal referral differences^5^ based partly on cultural differences and the concept of “elderly” being not identical worldwide. Therefore, in parallel to exploring biological DMR mechanisms, it is also essential to better understand referral patterns, both in AsI patients and in EAI patients, to minimize undertreatment of DMR worldwide.^3^

### DMR management and outcomes

A remarkable finding of our study is that AsI patients enjoy similarly high rates of mitral repair and similarly good postintervention survival as EAI patients with similar DMR definition, robustly supported by landmark analysis showing similarly high early repair survival benefit. Hence, there is no doubt that early repair benefit is of similar and attractive magnitude in both populations and that DMR undertreatment in our AsI patients is strongly linked to overall excess mortality. Indeed, our study demonstrates for the first-time differences in DMR management, with mitral intervention rates being much lower/delayed in AsI patients, with considerable gap within the first year, only minimally attenuated later. Ultimately, AsI patients incur excess mortality vs EAI patients in the long term after diagnosis. Previous long-term cohorts demonstrated that, apart from patients with heart failure symptoms, excess mortality takes many years to become significant,^1^, ^2^, ^3^, ^4^ explaining the very progressive benefit observed with early surgery,^9^ similar to our study. Thus, it is not surprising that restoration of life expectancy by mitral surgery in EAI patients and excess mortality related to aging and undertreatment in AsI patients yield a survival curve crossing after several years of follow-up, emphasizing the importance of long-term follow-up to uncover management consequences. Indeed, it is quite remarkable that including early surgery in survival models yields a notable reduction of excess mortality hazards in Asians, meaning that excess mortality in our AsI vs EAI cohorts is mostly explained by management differences (early mitral intervention). While comorbidity-linked mortality may be a concern, lower EuroSCORE II in AsI patients, similar postoperative survival, and persistent excess mortality in AsI patients after matching for comorbidities strongly argue against this concern. Thus, in our study all data, adjusted and matched analyses converge toward linking persistent excess mortality among AsI patients and their notable DMR undertreatment. While geographical deficiencies in cardiac surgery availability exist,^26^ these are mostly affecting low-income countries, and are not relevant to Hong Kong and Singapore, making this potential explanation unlikely.

Other determinants may affect lower interventional rates. Clinical guidelines during the study^6^^,^^7^ placed as Class I indication for DMR surgery, an enlarged absolute LVESD based on its association with poor outcome under medical management.^27^ Surprisingly, these remain unchanged, while guidelines recommend normalization to body surface area for aortic regurgitation. Our data show that reaching such absolute thresholds is less likely in AsI patients than EAI patients. Furthermore, LV and LA size are also integral part of grading DMR severity, and smaller dimensions may suggest less severe regurgitation.^21^ Lack of accounting for body size not only is relevant to countries with generally smaller body sizes, but also affects women with DMR in Western countries, who also are misjudged by absolute diameters not adjusted to body size, minimizing early referral to surgery, and resulting in worse outcomes vs men.^10^^,^^28^ Hence, accounting for body size during DMR imaging is crucial in all populations, particularly in view of the demonstrated link of LVESD corrected for body surface area (≥22 mm/m^2^) to outcome.^11^ This would represent a first step in avoiding underestimation of volume overload in DMR and in using appropriate imaging references.

Hesitancy in taking the immediate risk, even small, of mitral surgery/intervention in AsI patients may be heightened without declared symptoms^27^ and atrial fibrillation,^29^ while benefits of early surgery may occur quite later. Hesitancy may also be emphasized by hypertension and diabetes, which create confusion on causes of cardiac remodeling.^30^ Whether cultural differences, reluctance to intervene before clinical complications, and a framework of primum non nocere^31^ are operative in DMR undertreatment is difficult to prove, but they are crucially present in our experience. We observe that Asians are likely to attribute symptoms to the “normal aging process” and may hide those for fear of aggressive treatment. Asians may also seek alternative traditional medicine or acupuncture as first line and may consider “Western” medicine as a last resort.

It took considerable academic efforts to emphasize early surgery importance for DMR in Europe and North America,^32^ and to minimize cultural beliefs affecting undertreatment.^3^ Educational efforts for consideration of mitral interventions by AsI patients and physicians warrants further attention. It is also possible that percutaneous/minimally invasive DMR treatment may contribute to better acceptance of early DMR repair.

### Study strengths and limitations

The study is first to demonstrate differences in long-term outcomes in AsI patients vs EAI patients with DMR diagnosed similarly and reliably by echocardiography, but origin is not a randomizable exposure and does not come in isolation. Furthermore, ethnic/racial data abstraction is unlawful in continental Europe and is not part of our study. Hence, routine practice of institutions in various geographic locations is the only approach to provide insights into imaging, management, and outcomes in various world regions. Our study aimed to not portray therapeutic strategies and outcomes across the entire Asian continent, as rendering true diversity and complexity of the health care landscape in Asia would be impossible. We examined patients diagnosed with DMR due to flail leaflet in Hong Kong and Singapore, comparing them with those enrolled in the largest database with similar DMR definition, routinely managed in EAIs. In our cohorts, similar socioeconomic status and similar training of most colleagues created many similarities within routine practices, reflected by similarity of mitral repairability and postoperative outcomes. However, in observational multicenter studies, findings may be influenced by practices of involved institutions. Differing comorbidities in different continents may be a concern. However, similar postintervention survival and similar benefit of early repair strongly argues for lacking unsuspected excess noncardiac mortality among the AsI cohort. Short of studies covering entire countries, it is difficult to absolutely affirm geographical differences, but such countrywide studies have not been initiated to our knowledge. Delays in surgical intervention may be linked to different waiting times in different health care systems. Therefore, additional research is warranted to comprehensively investigate whether results are attributable to institutional or regional practices vs reflecting genuine geographical/cultural disparities.

Small body size and Asian origin are colinear, questioning the “real” independent determinant of AsI and EAI patients’ differences. Stratification by respective median body surface area showed that small body size associated univariately with less mitral intervention (HR: 0.77; 95% CI: 0.68-0.87; *P* < 0.0001). However, with comprehensive adjustment (as per Table 2), small body size became insignificant (*P* = 0.16), while the association of AsI patients with undertreatment remained highly significant (adjusted HR: 0.49; 95% CI: 0.42-0.58; *P* < 0.0001). DMR quantitation is not possible in all patients, and integrative DMR grading using all signs/measures is recommended, particularly with all patients carrying flail mitral leaflet, a specific sign for severe DMR.^21^ Yet, DMR quantitation, when performed, was quite similarly consistent with severe DMR, and is reassuring with regard to DMR severity in both cohorts.

## Conclusions

In 2 large cohorts from AsIs, we observed that patients present with similar features of mitral valve lesions and DMR severity compared with a large cohort of EAI patients of similar socioeconomic environment. However, in AsI patients, imaging may underestimate volume overload due to smaller cardiac cavities related to smaller body size, demonstrating the importance of accounting for body size in assessing cardiac enlargement.

Repair interventions predominate similarly in AsIs and EAIs and provide comparable survival benefit when performed early after diagnosis. Notwithstanding, management is very different, with fewer and delayed mitral interventions in AsI patients compared with EAI patients despite the comparable socioeconomic background. Consequently, the outcome is ultimately affected, with excess mortality in our cohort of AsI patients even after matching for all clinical differences. Although the population may not represent the entire Asian continent, or the full spectrum of hospitals and diverse practices of a multifaceted geographical region, these differences in DMR imaging/management/outcome, analyzed for the first time in the present study, show that it is crucial to consider similar early mitral interventions and to achieve optimal outcomes in patients with DMR worldwide. Further studies are needed, involving various institutions within Asia to assess the generalizability of these results across diverse populations, given the significant impact of economic factors on health care outcomes.

Perspectives **COMPETENCY IN PATIENT CARE AND PROCEDURAL SKILLS:** AsI patients with DMR have smaller bodies and, despite similar overload, display smaller hearts on imaging using absolute cardiac dimensions compared with EAI patients. Not accounting for body size deeply underestimates DMR-linked overload and treatment requirement. The same survival benefit is obtained from early mitral interventions for DMR in AsI patients compared with EAI patients. However, AsI patients with DMR are conservatively managed with fewer mitral interventions and subsequent reduced survival after DMR diagnosis. Addressing local cultural hindrance to mitral interventions is crucial to improving DMR outcome worldwide.

**TRANSLATIONAL OUTLOOK:** AsI patients present at a younger age for undefined reasons, biological vs referral. Future genetic and population-based studies should evaluate whether the development of myxomatous mitral degeneration is different in various populations and whether management modulations are in order.

## Funding Support and Author Disclosures

Dr Enriquez-Sarano has received personal fees from Edwards LLC, ChemImage Inc, and CryoLife Inc, outside the submitted work. Dr Ding has received personal fees from GE Healthcare; and nonfinancial support from Phillips Healthcare, outside the submitted work. Dr Lee has received grant support from Abbott, outside the submitted work. All other authors have reported that they have no relationships relevant to the contents of this paper to disclose.
