# Supplementary material for: Degenerative Mitral Regurgitation Outcomes in Asian Compared With European-American Institutions
Source: JACC Asia. 2024 May 21;4(6):468–80. doi: 10.1016/j.jacasi.2024.03.003 (PMC11291393; doi:10.1016/j.jacasi.2024.03.003)
Supplement: Supplemental Material [file mmc1.docx]

**SUPPLEMENTAL APPENDIX**

**List of the MIDA investigators**

**Mayo Clinic, Rochester MN, USA.** Giovanni Benfari, Benjamin Essayagh, Clemence Antoine, Joseph F Malouf, Hector Michelena, Vuyisile T Nkomo, Maurice L. Enriquez-Sarano

**Marche Polytechnic University, Ancona, Italy.** Federico Guerra, Alessandro Barbarossa, Antonio Dello Russo

**The Chinese University of Hong Kong.** Randolph Wong, Song Wan, Josie Chow, Yiting Fan, Alex P W Lee

**National Heart Centre Singapore, Singapore.** See Hooi Ewe, Khung Keong Yeo, Yann Shan Keh, Nadira Hamid, Ding Zee Pin

**University of Amiens, France.** Faouzi Trojette, Gilles Touati, Jean Paul Remadi, Henri J Poulain, Christophe Tribouilloy

**University of Bologna, Italy.** Raffaello Ditaranto, Giuseppe Caponetti, Carlo Savini, Davide Pacini, Elena Biagini

**University Campus-Bio-Medico of Rome, Italy.** Massimo Chello, Annunziata Nusca, Rosetta Melfi, Gian Paolo Ussia, Francesco Grigioni

**Université Catholique de Louvain, Belgium.** Jamila Boulif, Christophe de Meester, Gebrine. El Khoury, Bernhard L Gerber, Siham Lazam, Agnès Pasquet, Philippe Noirhomme, David Vancraeynest, Jean-Louis Vanoverschelde

**University of Marseille, France.** Frédéric Collart, Alexis Théron, Jean Francois Avierinos.

**University of Modena, Italy.** Andrea Barbieri, Francesca Bursi, Francesca Mantovani, Maria Grazia Modena, Giuseppe Boriani

**University of Milan, Italy.** Francesca Bursi

**University of Verona.** Giovanni Benfari, Andrea Rossi, Francesco Onorati, Flavio L. Ribichini

**SUPPLEMENTAL METHODS**

**Study population**

The inclusion/exclusion criteria of the AsI cohort and the MIDA registry were similar and have been previously described. General eligibility criteria were 1) Transthoracic echocardiographic diagnosis of DMR due to flail leaflets; 2) availability of comprehensive clinical/instrumental evaluation at baseline echocardiographic diagnosis; 3) exclusion of ischemic MR (including papillary muscle rupture); 4) isolated DMR without moderate or severe concomitant aortic valve disease, pericardial/congenital diseases, mitral stenosis or prior valve surgery; 5) absence of denial of research authorization. Patients who underwent mitral surgery based on outside echocardiograms without institutional full imaging were excluded.

**Echocardiography**

Transthoracic echocardiography was performed in routine clinical practice in each academic center and measurements were guided by American Society of Echocardiography guidelines as previously described (Supplemental Material). The diagnosis of flail leaflet was based on failure of leaflet coaptation with rapid systolic movement of the involved leaflet tip within the left atrium. Left ventricular (LV) and left atrial (LA) dimensions were assessed from parasternal views by 2D-guided linear or M-mode measurements at end-diastole and end-systole. LV ejection fraction (LVEF) was then calculated or estimated visually. DMR severity was assessed integratively as recommended by guidelines, including with DMR quantitation when necessary. MR was graded on a case by case basis by each investigator according to society guidelines. When possible, MR was quantified using the proximal isovelocity surface area method. This method, the vena contracta method and all signs and measures were integrated into the final DMR grading, most generally severe as expected with DMR due to flail leaflets.

Hemodynamics measured right-ventricular-systolic-pressure (RVSP) using tricuspid regurgitant velocity by continuous-wave Doppler and estimated right-atrial pressure.

**Statistical analysis**

To construct survival curves state of the art methods were used. For the age-adjusted survival after diagnosis compared between AsI and EAI the SAS software was used, specifically the macro “%newsruv”. The curve was obtained using Method=Direct. To create survival curves of the matched cohorts conventional Kaplan-Meier method was used. Because after matching age is similar between the matched sub-cohorts, there was no adjustment required in this analysis ^1,2^.

**SUPPLEMENTAL RESULTS**

**Propensity matched comparison of Asian and European American institutions patients.**

To balance baseline differences in clinical characteristics between patients from Asian and European and American centers, a propensity-score matching algorithm was performed to define sub-cohorts with similar baseline characteristics. The dependent variable of the logistic analysis used to construct the propensity score was AsI vs EAI status. For AsI patient, a control patient from EAI was selected (1:1) defined by the parameters using the greedy nearest neighbor propensity-score matching algorithm with 0.1 standard deviation caliper. The matching covariates were age, sex, NYHA class, history of systemic hypertension, diabetes, atrial fibrillation, left ventricular ejection fraction and EuroScore II. Categorical variables were not standardized and were used as they were.

With this matching we were able to obtain two populations of 434 patients each equalized for clinical characteristics, particularly age. The Supplemental Table S1 shows the distribution of characteristics in the matched populations.

The success of propensity-matching was assessed with standardized differences of covariates, (absolute standardized difference<10% indicated small imbalance) as shown below in the Supplemental Figure 1, and by directly comparing distributions in matched sub-cohorts as shown in supplemental Table 1.

**Supplemental Figure 1:** Propensity-matching characteristics before (purple squares) and after the matching (green circles).

AFIB Hx, history of atrial fibrillation; CAD HX, history of coronary artery disease; EF, ejection fraction; NYHA, New York Heart Association; Obs, observations.


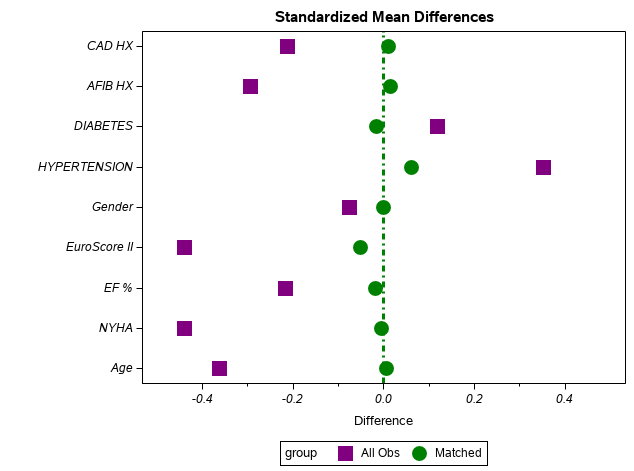


**Supplemental Table 1.** Propensity matched comparison of Asian and European American institutions patients.

|  | **Matched AsI and EAI comparison** | | |
| --- | --- | --- | --- |
|  | **AsI-patients**  **N=434** | **EAI-patients**  **N= 434** | **P Value** |
| *Clinical data* | | | |
| Age at diagnosis, years | 62.8±12.5 | 62.4±12.7 | 0.66 |
| Men, % | 73.5% | 73.5% | 1 |
| NYHA class, median [IQR] | 1[1-2] | 1[1-2] | 0.86 |
| Asymptomatic | 62% | 55% | 0.05 |
| Sinus rhythm at diagnosis | 78.8% | 77.2% | 0.57 |
| History of atrial fibrillation | 25.8% | 24.7% | 0.70 |
| Hypertension | 42.9% | 41.7% | 0.73 |
| Diabetes mellitus | 7.6% | 7.8% | 0.90 |
| Dyslipidemia | 30% | 34% | 0.18 |
| History of CAD | 26% | 24% | 0.58 |
| Euroscore II, % | 0.96±0.62 | 0.95±0.57 | 0.86 |
| *Morphometric data* | | | |
| Weight, kg | 64±17 | 82±19 | <0.0001 |
| Height, cm | 163±9 | 174±10 | <0.0001 |
| Body surface area, m^2^ | 1.69±0.24 | 1.96±0.24 | <0.0001 |
| LA diameter, mm | 48±12 | 48.6±5 | 0.26 |
| Indexed LA diameter, mm/m^2^ | 29±8 | 25±4 | <0.0001 |
| LVEDD, mm | 52±8 | 58±7 | <0.0001 |
| Indexed LVEDD, mm/m2 | 31.4±6.0 | 30.0 ± 4 | <0.0001 |
| LVESD, mm | 33.5±8.6 | 37±7 | <0.0001 |
| Indexed LVESD, mm/m^2^ | 20.2±5.7 | 19.0±3.5 | <0.0001 |
| LV EF, % | 62.9±7.5 | 62.9±8.7 | 0.97 |
| LV mass, g | 211±99 | 256±75 | <0.0001 |
| Indexed LV mass, g/m^2^ | 126±55 | 129±32 | 0.30 |

AsI, Asian Institution; CAD, coronary artery disease; EAI, European American institution; EF, ejection fraction; IQR, inter-quartile range; LA, left atrium, LV, left ventricular; LVEDD, left ventricular end diastolic diameter, LVESD, left ventricular end systolic diameter.

The matched populations were thus similar for the distribution of all clinical data as all clinical characteristics (even those not matched for) displayed no significant differences (Supplemental Table S1), while morphometric data showed the expected persistent body-size and absolute heart size differences (bottom Supplemental Table S1) between matched AsI and EAI sub-cohorts. Furthermore, similarly to the overall cohorts, matched cohorts showed in AsI-patients vs. EAI-patients larger cardiac size after normalization to body-surface-area (bottom Supplemental Table S1).

Also, similarly to the overall cohorts, matched cohorts displayed considerable differences in outcome. Indeed, despite the similar age, EuroScoreII, symptoms, LVEF, atrial fibrillation and comorbid conditions, AsI-patients incurred lower incidence of treatment by mitral interventions, (Supplemental Figure S2) with 1- and 5-year rates (57±2 and 67±2%) lower than EAI-patients (80±2 and 83±2%, P<0.0001) and incurred low HR of mitral interventions (0.57[0.49-0.67], P<0.0001) vs. EAI-patients, similarly to the overall cohorts.

When we analyzed these equalized populations resulting from this extensive matching, AsI patients were less referred to mitral interventions and, if operated, this occurred later compared to matched EAI-patients, p<0.0001 (Supplemental figure S2).

**Supplemental Figure 2:** Cumulative incidence of mitral valve intervention in matched As-I vs EA-I after the diagnosis of degenerative mitral regurgitation due to flail leaflets

Rates of mitral intervention after diagnosis indicated over the first 5 years of follow-up.

AsI, Asian Institution EAI, European American institution


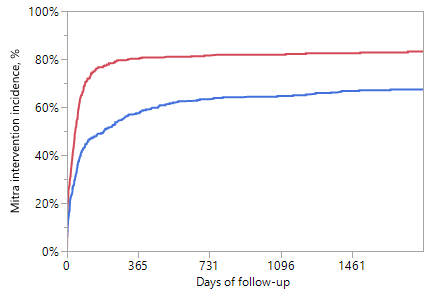


EAI patients

AsI patients

P<0.0001

Furthermore, in this matched analysis, despite the tight matching, particularly for age, AsI-patients incurred higher long-term mortality, (Supplemental Figure S3) with 10-year survival rates (61±5%) lower than EAI-patients (83±3%, P=0.018) and high HR of mortality (1.56[1.08-2.22], P=0.019) vs. EAI-patients, similarly to the overall (unmatched) cohorts.

The survival after DMR diagnosis of these matched sub-cohorts of similar age, was similar during the first 5 years of follow-up but secondarily, high-mortality rates observed in matched AsI-patients yielded overall excess mortality p=0.018. (Supplemental Figure S3)

**Supplemental Figure 3**: Survival analysis (direct comparison) in matched As-I vs EA-I after the diagnosis of DMR due to flail leaflets


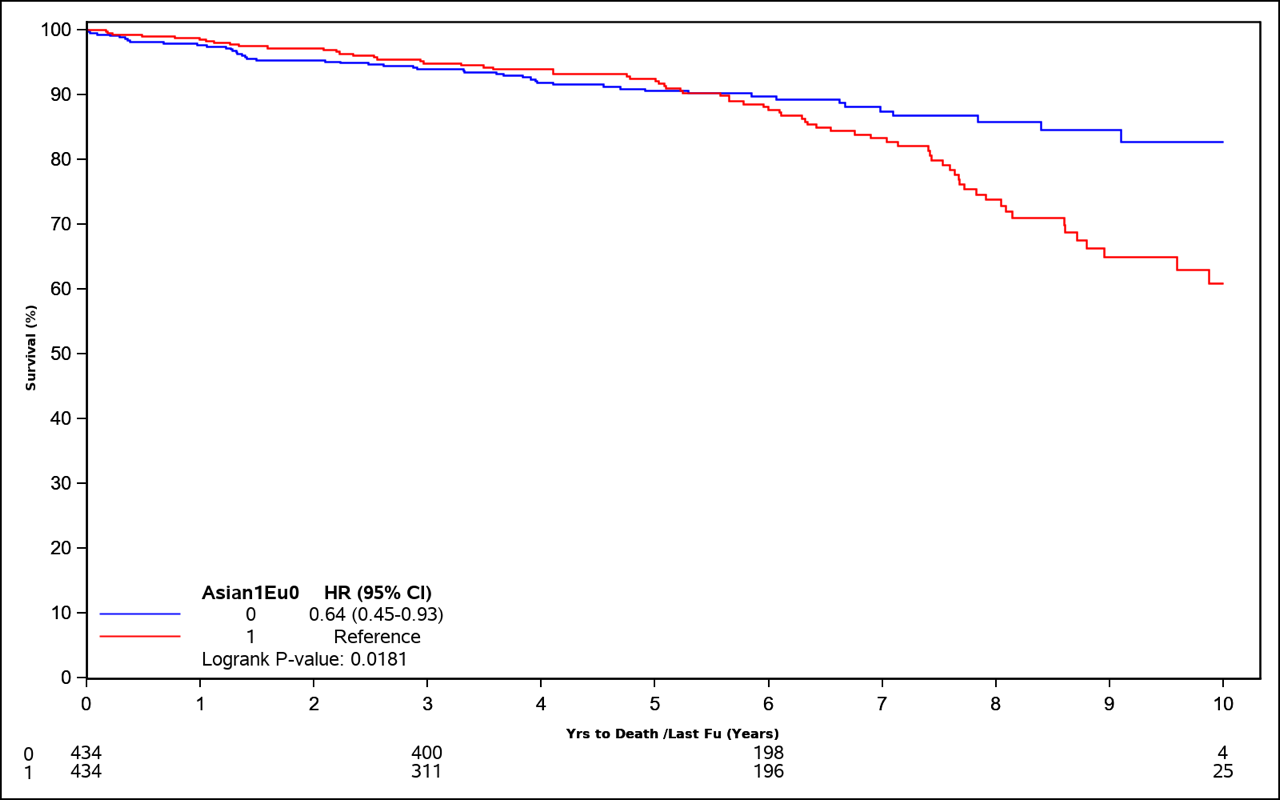


Note that in these matched sub-cohorts, the observed survival and the differences between Asi-patients and EAI-patients are almost identical to those observed in the age-adjusted survival curves presented in the main manuscript.

**Characteristics by early surgery**

Because clinical characteristics may influence the decision to withhold early surgery and favor initial medical management, we addressed this specific issue in Supplemental Table S2.

**Supplemental Table 2**

|  | **ASI Cohort** | | **P** | **EAI Cohort** | | **p** |
| --- | --- | --- | --- | --- | --- | --- |
|  | **Initial conservative management**  **N 428** | **DMR correction within 3 months**  **N 309** |  | **Initial conservative management**  **N 223** | **DMR correction within 3 months**  **N 459** |  |
| Age | 62±1 | 59 ±1 | <0.001 | 70±1 | 64±1 | <0.001 |
| Men | 69% | 72% | 0.280 | 75% | 72% | 0.44 |
| NYHA class I | 77% | 70% | 0.003 | 49% | 41% | 0.22 |
| NYHA class II | 17% | 16% |  | 36% | 44% |  |
| NYHA class III | 5% | 12% |  | 12% | 12% |  |
| NYHA class IV | 1% | 2% |  | 3% | 3% |  |
| Sinus rhythm at diagnosis | 85% | 80% | 0.100 | 71% | 68% | 0.45 |
| History of CAD | 17% | 29% | <0.001 | 28% | 33% | 0.45 |
| EuroScore II | 0.93±0.02 | 0.85±0.03 | 0.054 | 2.03±0.11 | 1.17±0.08 | <0.001 |
| Severe mitral regurgitation | 80% | 94% | <0.001 | 78% | 95% | <0.001 |
| Isolated posterior Flail | 85% | 86% | 0.540 | 76% | 80% | 0.24 |
| EF, % | 61±9 | 62±9 | 0.92 | 62±11 | 64±7 | 0.003 |
| LVESD, mm | 33±8 | 34±9 | 0.41 | 36±8 | 37±6 | 0.33 |

Abbreviations like in Supplemental Table 1.

In both groups of AsI and EAI older age and lower DMR severity were associated with the choice of initial medical management, whereas the cardiac rhythm, EF and LV ESD do not appear to play a significant role in both cohorts. However, there are also differences, whereby symptoms are much more linked to the decision to early intervene in AsI patients than in EAI, whereas the risk of the intervention (in older patients) interferes much more with the intervention decision in EAI than AsI patients. At the individual level these reasons are not clearly expressed by the physicians managing the patients, as well as the patients’ wishes so that we should be prudent in our interpretation.

References

1. Borgan Ø. Modeling survival data: extending the cox model. Terry M. Therneau and Patricia M. Grambsch, Springer-Verlag, New York, 2000. ISBN 0-387-98784-3. Stat Med (2001) 20(13):2053–4. doi: 10.1002/sim.956

2. Therneau TM, Crowson CS, Atkinson EJ. 2015. Adjusted Survival Curves. https://cran.r-project.org/web/packages/survival/vignettes/adjcurve.pdf.
